# Evaluating the Equilibrium Association Constant between ArtinM Lectin and Myeloid Leukemia Cells by Impedimetric and Piezoelectric Label Free Approaches

**DOI:** 10.3390/bios4040358

**Published:** 2014-10-03

**Authors:** Fernanda C. Carvalho, Denise C. Martins, Adriano Santos, Maria-Cristina Roque-Barreira, Paulo R. Bueno

**Affiliations:** 1 Department of Physical Chemistry, Chemistry Institute, São Paulo State University (UNESP), 55 Prof. Francisco Degni Street, 14800-060, São Paulo, Brazil; E-Mails: nandaferdecarvalho@gmail.com (F.C.C.); denisecm86@gmail.com (D.C.M.); santosadriano29@gmail.com (A.S.); 2 Department of Cellular and Molecular Biology and Pathogenic Bioagents, Ribeirão Preto Medical School, University of São Paulo (USP), 3900 Bandeirantes Avenue, 14049-900, Ribeirão Preto, São Paulo, Brazil; E-Mail: mcrbarre@fmrp.usp.br

**Keywords:** ArtinM, lectin, myeloid leukemia cells, electrochemical impedance spectroscopy, quartz crystal microbalance, Langmuir isotherm, equilibrium association constant (*K_a_*)

## Abstract

Label-free methods for evaluating lectin–cell binding have been developed to determine the lectin–carbohydrate interactions in the context of cell-surface oligosaccharides. In the present study, mass loading and electrochemical transducer signals were compared to characterize the interaction between lectin and cellular membranes by measuring the equilibrium association constant, *K_a_*, between ArtinM lectin and the carbohydrate sites of NB4 leukemia cells. By functionalizing sensor interfaces with ArtinM, it was possible to determine *K_a_* over a range of leukemia cell concentrations to construct analytical curves from impedimetric and/or mass-associated frequency shifts with analytical signals following a Langmuir pattern. Using the Langmuir isotherm-binding model, the *K_a_* obtained were (8.9 ± 1.0) × 10^−5^ mL/cell and (1.05 ± 0.09) × 10^−6^ mL/cell with the electrochemical impedance spectroscopy (EIS) and quartz crystal microbalance (QCM) methods, respectively. The observed differences were attributed to the intrinsic characteristic sensitivity of each method in following Langmuir isotherm premises.

## 1. Introduction

Alteration of cell-surface glycosylation is a universal feature of cancer cells and is a major contributor to the development of malignancy [1]. Lectins are carbohydrate-binding proteins or glycoproteins of non-immune origin that recognize and reversibly bind to glycans without altering their covalent structure. Plant lectins are important tools in cell biology and immunology research with a potential for clinical application [2]. In particular, plant lectins can be used to identify glycan determinants that are markers of clinical interest and may possess anti-tumor properties [1]. One such example is ArtinM, a mannose-binding lectin from the seeds of *Artocarpus heterophyllus*.

ArtinM is a tetrameric, non-glycosylated protein composed of identical 16-kDa protomers, each of which has a carbohydrate-recognition domain that exhibits high specificity for the trimannoside Manα1-3[Manα1-6]Man. It is present in the core of N-glycans attached to receptors on the cell surface [3,4]. ArtinM possesses many relevant biological properties such as inflammatory-cell activation [5], Th1 immunomodulation [6], and tissue regeneration [7,8]. In addition, ArtinM promotes the cell death of NB4 promyelocytic acute leukemia cells by recognition of β1,6-GlcNAc-branched N-glycans (aberrant glycosylation) on cell surface [9]. 

The affinity parameters of the lectin–carbohydrate interaction have been proposed using label-free techniques, including surface plasmon resonance [10,11,12], piezoelectric [13,14,15,16,17,18,19] and impedimetric approaches [20,21]. Formally, the affinity interactions between a receptor and multiple sites in a ligand is an avidity interaction [12,22] and the observed *K_a_* is an averaged “equilibrium association constant”. The onset of an avidity interaction would be observed as a departure from the Langmuirian kinetic time course indicating at least two kinetic processes [12,23].

The quartz crystal microbalance (QCM) was initially employed in analytical chemistry to measure the mass bound to a quartz sensor in gas phase, with a decrease in the oscillating frequency of the quartz crystal denoting an increase in the mass adsorbed to the sensor surface, following the Sauerbrey equation [24,25]:

(1) $$\Delta f=-\frac{f_{0}^{2}}{F_{q}\rho_{q}A_{el}}\Delta m$$

where Δf is the frequency shift (Hz), Δm is the adsorbed mass (g), $f_{0}$ is the fundamental frequency of the QCM crystal (Hz), $\rho_{q}$ is the density of quartz (2.648 g/cm^3^), $F_{q}$ is the shear modulus of quartz for an AT-cut crystal (2.947 × 10^11^ g/cm·s^2^), and $A_{el}$ is the piezoelectrically active crystal area (cm^2^). The development of QCM for use in liquids is based on the fact that the part of the vibration energy that is dissipated (∆D) due to viscoelastic characteristics is mainly prominent in the biosensory interface in liquid media. Therefore, it is useful to evaluate the ∆D/∆f ratio, which should be smaller than 0.2 × 10^−6^/Hz, as this is the highest limit at which the Sauerbrey equation can be quantitatively applied [26,27].

Despite the ability for QCM to measure binding events, electrochemical approaches such as electrochemical impedance spectroscopy (EIS) offer the most suitable tool owing to their high levels of sensitivity and miniaturization within a multiplex capability. The complex impedance $Z^{*}$ is the quotient of the voltage–time perturbation and the resulting current-time response function [28]. The use of an EIS transducer signal in biological sensorial applications is based on the premise that an interaction between a biological receptor and its target species recruited from the solution causes a change in the interfacial electron transfer kinetics between a redox probe in the solution and metallic electrode sites. Therefore, the transducer signal in EIS represents the electrochemical impediment of the redox probe caused by the target binding, and it is measured by obtaining the charge-transfer resistance ($R_{ct}$) from the EIS spectra [28]. The use of EIS in measurement events related to a classic antigen–antibody binding model has been reported to show good reproducibility and sensitivity [29,30,31].

QCM and EIS techniques have also been used to characterize lectin–cell interactions, including cell-surface glycosylation [32,33] or discrimination among malignant stages of tumor cells [19,32,33,34]. In this study, we performed the first comparative evaluation of the sensitivity between piezoelectric (by QCM) and impedimetric (by EIS) approaches in determining lectin–cell interactions. In particular, the interactions between ArtinM lectin and NB4 leukemia cells were evaluated under Langmuir adsorption (or biochemistry-binding) assumptions. The NB4 leukemia cell line was chosen because of its known susceptibility to ArtinM death [9].

## 2. Experimental Section

### 2.1. Reagents and Cells

All reagents described in this section were purchased from Sigma-Aldrich.

The lectin ArtinM used in this work was extracted from *Artocarpus heterophyllus* seeds by affinity chromatography to immobilized d-mannose, as previously described [35].

All the solutions used in the analytical procedures were prepared with Milli-Q-purified water (Millipore) with conductivity of 18.2 MΩ·cm at 25 °C. The protein solutions were prepared and used in 10 mM phosphate-buffered saline (PBS; pH 7.4).

The promyelocytic acute leukemia cell line NB4 was cultured in RPMI 1640 medium supplemented with 10% heat-inactivated fetal bovine serum and streptomycin/penicillin (100 µg/mL; Gibco), and incubated at 37 °C in a humidified atmosphere containing 5% CO_2_. The culture media were changed every 48 h. A portion of the cells was collected and fixed with 2% paraformaldehyde at room temperature for 20 min, followed by a wash with 1% glycine. Cells were counted in a Neubauer chamber (Weber Scientific International) to adjust volumes to the required concentration.

### 2.2. Surface Engineering and Lectin Sensor Functionalization

The quartz used was 16 mm in diameter, AT-cut, and had a fundamental frequency of 5 MHz (polished quartz crystals were provided by the Q-SENSE company with gold electrodes). Prior to the experiments, the quartz electrode surface was cleaned and characterized [16]. For EIS, the gold electrode (2 mm; Metrohm) was cleaned and the surface area was determined [29].

**Figure 1:**
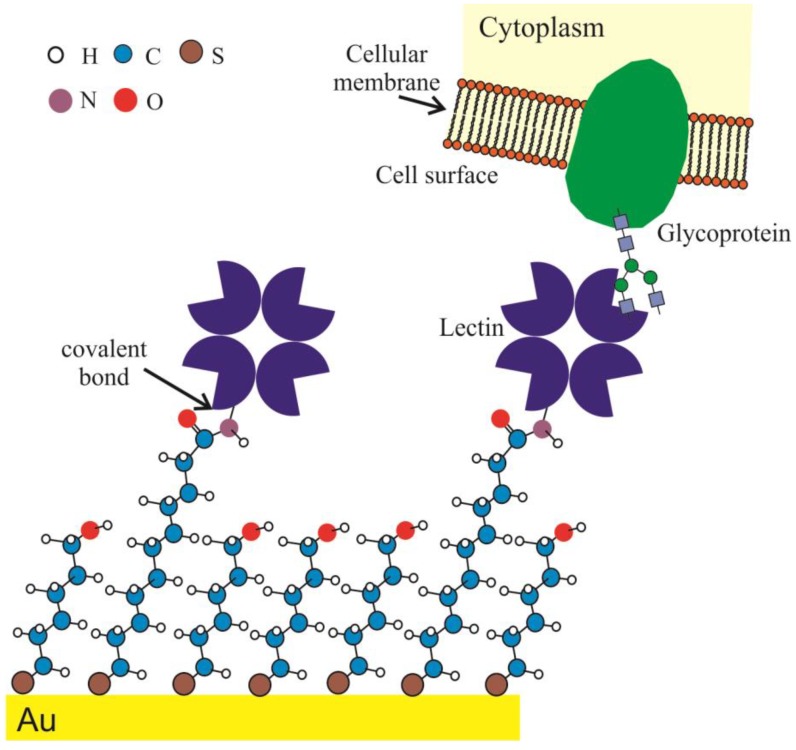
Schematic representation of functional SAM used to characterize ArtinM–cell binding by EIS and QCM. The surface was constructed onto a gold surface using mixed thiol–SAM structures, in which 11-mercaptoundecanoic acid served as a receptor-supportive layer for lectin attachment, and 6-mercaptohexanol served as a spacer layer. ArtinM immobilization was achieved through standard EDC/NHS bioconjugation, and the nonspecific sites were blocked with gelatin (not shown). NB4 cells at various concentrations were quiescently lying on the interface for EIS or were injected in flux for QCM. The schematic illustrates only one carbohydrate recognition domain of ArtinM interacting with only one N-glycan, the glycoprotein of the cell surface. Indeed, many N-glycans on glycoproteins of cell surface could be recognized by all carbohydrate recognition domains of ArtinM. H: hydrogen, C: carbon, S: sulfur, N: nitrogen, O: oxygen.

The strategy adopted for the comparative analysis of the EIS and QCM approaches was based on the construction of a self-assembled monolayer (SAM) that was made with a 1:25 mixture of 2 mmol/L 11-mercaptoundecanoic acid (Sigma), as an anchor, and 2 mmol/L 6-mercaptohexanol (Sigma), as a spacer, in 200 μL ethanol. The electrode was washed first in pure ethanol and then in deionized water and dried under nitrogen. ArtinM (0.15 mg/mL solution; 300 μL for QCM and 30 μL for EIS) immobilization was achieved through standard EDC/NHS bioconjugation chemistry and then anchored to the electrode surface; a solution of 0.4 mol/L EDC (N-(3-Dimethylaminopropyl)-N′-ethylcarbodiimide hydrochloride) and 0.1 mol/L NHS (N-Hydroxysuccinimide) was prepared and added to the electrode, thereby activating the terminal carboxyl groups on the thiol for 30 min. The remaining NHS esters were deactivated by the addition of a 1 mol/L ethanolamine solution for 5 min, and the surface was thoroughly rinsed in deionized water. A 0.1% gelatin solution was added for 30 min to block non-specific sites (Figure 1).

### 2.3. Electrochemical Impedance Spectroscopy (EIS) Analysis

EIS measurements were carried out on an AUTOLAB potentiostat (model PGSTAT30) that was controlled by the NOVA program, which was used for all electrochemical measurements. A three-electrode setup was used for all procedures, consisting of a 2.0 mm diameter gold working electrode (Metrohm), a platinum mesh counter electrode, and an Ag|AgCl, 3 mol/L KCl reference electrode.

EIS was used for characterization of the all-building receptive surface steps and binding analysis. EIS measurements were recorded in the presence of 1 mmol/L [Fe(CN)_6_]^3−/4−^ as the redox probe in a KNO_3_ 1 mol/L as a supporting electrolyte, and were conducted in a frequency range of 0.01 Hz to 1 MHz, in the fixed potential of ~0.22 V, and at an amplitude of 10 mV (peak to peak). After electrochemical characterization of the functionalized electrode, NB4 aliquots (30 µL) were quiescently lying on the interface (Figure 1) with concentrations ranging from 1 × 10^3^ to 1 × 10^6^ cells/mL in PBS for 35 min, followed by EIS analysis at the same conditions used for the SAM functionalization process. All measurements were performed in triplicate. Subsequently, $R_{ct}$ was measured by approximation based on calculating the diameter of the semi-circle of the real part of impedance (*Z*').

### 2.4. Quartz Crystal Microbalance (QCM) Analysis

A Q-SENSE E4 QCM system with an ISMATEC IPC peristaltic pump was used for QCM measurements. The crystal was then placed in the QCM chamber, and PBS was used as a carrier stream with a flow rate of 100 µL/min. After stabilization of quartz crystal frequency oscillation, the NB4 cells (300 µL) were injected at concentrations of 1 × 10^5^ to 1.5 × 10^7^ cells/mL, and the flow was turned off for 35 min (Figure 1). Thereafter, the flow was restarted to remove any non-interacted cells, and the Δf value was obtained (all Δf values are reported in this work as modulus values). All measurements were performed in triplicate.

## 3. Results and Discussion

### 3.1. Langmuir Isotherm and $K_{a}$ Determination

The equilibrium association constant ($K_{a}$) between a target (*i.e.*, NB4 cells) and a receptor bound to the sensor surface (*i.e.*, ArtinM lectin) can be readily obtained by using the Langmuir adsorption isotherm model [16,29]. The 1:1 interaction model was used as previously described [16,32], even with multiple sites of interaction on cells, and more than one site of interaction on ArtinM. Consequently, $K_{a}$ represents herein an average of lectin–multiple sites interaction.

In the present case, the biological equilibrium association constant, $K_{a}$, between ligands can be theoretically obtained by varying the site surface occupation, Γ, at a given cell bulk concentration [ ].

There are two mutually exclusive possible scenarios using this approach: unoccupied ArtinM-free sites: (ArtinM) and occupied (ArtinM·Cell) receptor sites. The dynamic equilibrium between the lectin (ArtinM) and the lectin–cell complex (ArtinM·Cell), according to the Langmuir isotherm assumptions as described previously [16,28], is given by Equation (2):

(2) ArtinM+Cell⇌ArtinM·Cell

The affinity constant, $\;K_{a}$, is then obtained as:

(3) $$K_{a}=\frac{\left[ArtinM\cdot Cell\right]}{\left[ArtinM\right]\left[Cell\right]}$$

where [ ] stands for concentration. Assuming a surface coverage occupation percentage θ and a percentage of available sites 1−θ, Equation (3) can be rewritten as:

(4) $$K_{a}\left[cell\right]=\frac{\theta }{1-\theta }$$

In solving Equation (4) for *θ*

(5) $$\theta =\frac{K_{a}\left[Cell\right]}{1+K_{a}\left[Cell\right]}$$

the surface coverage, Γ (number of cells per cm^2^), is proportional to θ. Therefore, it can be assumed that $\Gamma =\theta \Gamma_{m}$, where $\Gamma_{m}$ is the maximum amount of adsorption probability when θ approaches unity (note that $\Gamma_{m}={\left(∆R_{ct}\right)}_{m}$ in EIS and $\Gamma_{m}={\left(∆f\right)}_{m}$ in QCM). Thus, Γ can be expressed as:

(6) $$\Gamma =\frac{\Gamma_{m}K_{a}\left[Cell\right]}{1+K_{a}\left[Cell\right]}$$

Equation (6) can be linearized as:

(7) $$\frac{\left[Cell\right]}{\Gamma }=\frac{\left[Cell\right]}{\Gamma_{m}}+\frac{1}{K_{a}\Gamma_{m}}$$

Experimentally, Γ can be tracked (proportionally) by monitoring $R_{ct}$ in EIS or Δf (frequency shift) in QCM. Indeed, Equation (7) can be written in terms of the proportional variation of Γ to $\Delta R_{ct}$ or Δf and is suitably used to fit experimental data. In the specific case of the present study, plots of $\left[Cell\right]/\Delta R_{ct}\;$and [Cell]/Δf against [Cell] were constructed. The $K_{a}\;$value was then easily determined by calculating the quotient between the angular ($1/{\left(\Delta R_{ct}\right)}_{m}$ or $1/{\left(∆f\right)}_{m}$) and linear ($1/K_{a}{\left(∆R_{ct}\right)}_{m}$ or $1/K_{a}{\left(∆f\right)}_{m}$) coefficients of Equation (7).

### 3.2. Lectin Biosensor Construction 

It is important to note that in considering the Langmuir isotherm model to describe molecular adsorption on a surface (in gas or liquid phase), some premises are adopted: (i) the surface is homogeneous, (ii) all sites are energetically equivalent, (iii) each site can hold at most one molecule of adsorbate (mono-layer coverage only), and (iv) adsorbate molecules interacting with adjacent sites do not exist [15,16,24]. In order to conform to these premises, the surface chemistry architecture of the sensor was constructed using a mixed SAM composed of an anchored thiol and a spacer thiol. For both EIS and QCM ArtinM sensor-surface engineering, the lectin was immobilized by a covalent bond on the electrode surface using mixed SAM chemistry, as described in the Experimental Section. The monitoring of the ArtinM sensor fabrication process was analyzed independently for QCM and EIS.

**Figure 2:**
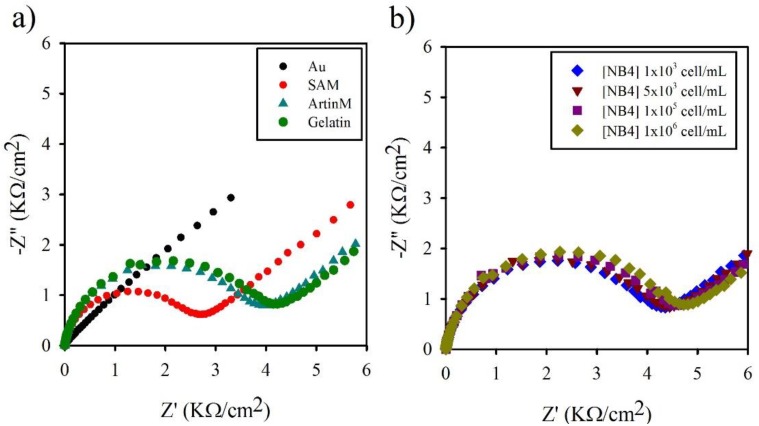
Determination of the ArtinM–NB4 interaction by EIS. Nyquist plots of impedance spectra were constructed by formation of the SAM, the immobilization of ArtinM, and blocking with gelatin (**a**), and by addition of different concentrations of NB4 cells (**b**). The impedance spectra were recorded in the presence of 1 mmol/L [Fe(CN)_6_]^3−/4−^ as the redox probe in a KNO_3_ 1 mol/L as a supporting electrolyte, and were conducted in a frequency range of 0.01 Hz to 1 MHz, in the fixed potential of ~0.22 V *vs*. Ag|AgCl, and at an amplitude of 10 mV. *Z*'' is the imaginary and *Z*' is the real part of impedance. $R_{ct}$ was measured from approximation of the diameter of the semi-circle of real part of impedance, *Z*'.

The construction of the EIS lectin sensor was monitored by using Nyquist plot diagrams, where the semicircles reflect charge transfer restrictions that are sterically or electrostatically imposed as the films are being constructed. In other words, this reflects the sharp increases in $R_{ct}$ as the receptor layer is fabricated. As shown in Figure 2, the SAM monolayer promoted a charge transfer restriction that increased after ArtinM coupling. A slightly smaller charge transfer restriction was promoted by the gelatin used to block reminiscent similar sites of interaction, indicating that the immobilization process was successful.

**Figure 3:**
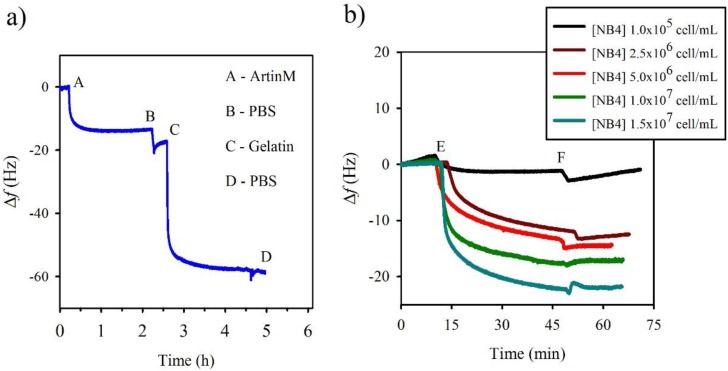
Determination of the ArtinM–NB4 interaction by QCM. (**a**) Real-time frequency shifts (*Δf*) shown as a function of measured time of adsorption of lectin by ArtinM: (A) the initial moment of ArtinM injection, (B) injection of PBS to wash the crystals, (C) injection of gelatin to block non-occupied sites, (D) re-injection of PBS for washing the non-adsorbed gelatin. (**b**) Real-time frequency shifts as a function of time for ArtinM interacting with different concentrations of NB4 cells: (E) initial moment of cell injection, (F) injection of PBS. The final values correspond to the frequency shift due to specific binding between the cells and ArtinM.

Although QCM lectin sensor construction was done quiescently, the real-time ArtinM functionalization was illustrated in the plot of Δf versus time (seconds). As shown in Figure 3a, the crystal frequency decreased, reflecting mass adsorption [25] upon lectin injection, confirming that ArtinM was immobilized on the transducer surface. Furthermore, the gelatin injection also produced a crystal frequency decrease, indicating successful blockage of nonspecific sites.

### 3.3. Comparison of $K_{a}$ Obtained by EIS and QCM

In the EIS analysis, the incubation of NB4 cells on the ArtinM sensor caused a change in the electron transfer resistance (Figure 2). The minimum (precision signal detection of the system) and maximum (saturation of the surface) detectable signals of NB4 cells with the ArtinM-functionalized electrode were examined by testing a range of NB4 concentrations. The specificity of ArtinM interaction was previously reported by N-Glycan depletion on NB4 cells [9], and by pre-incubation with specific sugar (mannose or Manα1-3[Manα1-6]Man) [36,37].

**Figure 4:**
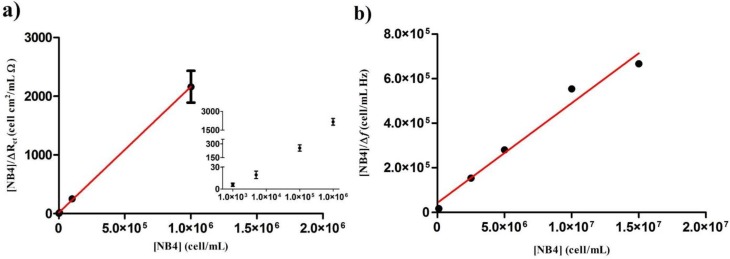
Linearized Langmuir isotherms for describing the ArtinM–NB4 interaction. The plots obtained by the EIS approach (**a**) and the QCM approach (**b**). The inset in (**a**) shows a log representation of cell concentration, R^2^ = 0.99. In (**b**), the values of SD are smaller than the point size and are shown in Table 2 (R^2^ = 0.97).

Different concentrations of NB4 cells (1 × 10^3^ to 1 × 10^6^ cells/mL) were employed to react with the ArtinM-receptive surface, and Langmuir isotherm curves were obtained by plotting the relative change of the $\Delta R_{ct}$ ($\Delta R_{ct}=\;R_{ct\;\left(cell\;concentration\right)}-\;R_{ct\;\left(gelatin\right)}$) value *versus* cell concentration (Table 1). The range of cell concentrations used was consistent with that adopted in previous cell impedimetric analyses [34]. The calibration curve associated with the $\left[Cell\right]/\Delta R_{ct}\;$ *versus* cell concentration plot (Figure 4a and Table 1) was used to obtain $K_{a}\;$from the slope of the curve, which was (8.9 ± 1.0) × 10^−5^ mL/cell.

**Table 1:** The electron transfer resistance of ArtinM–NB4 interaction.

[NB4] (cell/mL)	∆*R_ct_* (Ω/cm^2^)	[NB4]/∆*R_ct_* (cell·cm^2^/mL·Ω)
1 × 10^3^	186 ± 72	05.81 ± 2.25
5 × 10^3^	273 ± 83	19.33 ± 5.05
1 × 10^5^	397 ± 55	254.81 ± 34.05
1 × 10^6^	467 ± 54	2162.44 ± 270.96

On the other hand, in the QCM analysis, the range of cell concentrations used to obtain real-time response curves was 1 × 10^5^ to 1.5 × 10^7^ cells/mL. The crystal frequency decreased at the time of cell injection, indicating that the cells interacted with ArtinM that was immobilized on the transducer surface. After initiation of the PBS flux (at 3000 s), the difference between the stable original and final values corresponded to the frequency shift due to specific binding of the cells and ArtinM (Figure 4b and Table 2). In addition, the ∆D/∆f value (~0.012 × 10^−6^/Hz to 0.15 × 10^−6^/Hz) was smaller than the predicted value (0.2 × 10^−6^/Hz) as the highest limit to apply the Sauerbrey equation [27], excluding viscoelastic interference. Different concentrations of NB4 cells were employed to react with the ArtinM-receptive surface in order to construct the Langmuir isotherm curves, and the linear relationship [Cell]/∆f against [Cell] is shown in Figure 4b and Table 2. The $K_{a}\;$ value was obtained directly from the slope of these curves as (1.05 ± 0.09) × 10^−6^ mL/cell.

**Table 2:** Frequency shift variation of Artin-NB4 interaction.

[NB4]	Δ*f* (Hz)	[NB4]/Δ*f* (cell/mL·Hz) ×10^5^
1.0 × 10^5^	05.93 ± 0.89	0.17 ± 0.02
2.5 × 10^6^	16.39 ± 1.39	1.54 ± 0.13
5.0 × 10^6^	17.83 ± 0.07	2.80 ± 0.01
1.0 × 10^7^	18.05 ± 0.01	5.54 ± 0.01
1.5 × 10^7^	22.50 ± 0.01	6.67 ± 0.01

These results clearly demonstrate that the $K_{a}$ value depends on the methodology used (EIS or QCM). EIS has the advantage of requiring a lower volume (10 × lower) of analyte. A particularly remarkable observation of this study was that under the Langmuir premise, $K_{a}$ appears to be very sensitive to the range of cell concentration required in each technique—a low range for EIS and a saturation range for QCM—demonstrating that EIS (as an electrochemical technique) has higher sensitivity compared to QCM.

In summary, the results clearly demonstrate that affinity estimated through $K_{a}$ differs depending on whether EIS or QCM analysis is used for a lectin–cell binding model under Langmuir model premises. The capability of evaluating $K_{a}$ as demonstrated herein can be helpful in tumor cell characterization and in the study of drug delivery [32,33,34,38].

## 4. Conclusions

Label-free methods, e.g., EIS and QCM, have been developed for evaluating the lectin–cell interaction through the $K_{a}$ measurement. The methodological difference between EIS and QCM was reported herein, and the differences in $K_{a}$ values obtained between the methods were explained in accordance with Langmuir isotherm premises. Although the Langmuir premises are valid for both methodologies, specifically in the lectin–cell model, the EIS method showed better sensitivity since it requires a lower range of cells to transduce a binding signal, which is a significant advantage of the EIS approach over QCM. The determination of the equilibrium association constant in a lectin–cell model has a particular relevance for cellular glycoarray analyses and for the use of lectin as a marker for general tumor diagnostics and treatments.
